# Determinants of infant and young child feeding practices by mothers in two rural districts of Sindh, Pakistan: a cross-sectional survey

**DOI:** 10.1186/s13006-017-0131-z

**Published:** 2017-09-16

**Authors:** Gul Nawaz Khan, Shabina Ariff, Ubaidullah Khan, Atif Habib, Muhammad Umer, Zamir Suhag, Imtiaz Hussain, Zaid Bhatti, Asmat Ullah, Ali Turab, Ali Ahmad Khan, Alba Cecilia Garzon, Mohammad Imran Khan, Sajid Soofi

**Affiliations:** 1 Department of Paediatrics and Child Health, The Aga Khan University, Karachi, Pakistan; 2 Department of Paediatrics, King Edward Medical University, Lahore, Pakistan; 3 World Food Programme, Islamabad, Pakistan; 4 Center of Excellence in Women and Child Health, The Aga Khan University, Karachi, Pakistan

**Keywords:** Infant, Young, Child, Feeding, Practices, Factors, Pakistan

## Abstract

**Background:**

Infant and young child feeding (IYCF) practices during the first two years of life are important for the growth and development of a child. The aim of this study was to assess IYCF practices and its associated factors in two rural districts of Pakistan.

**Methods:**

A cross-sectional study was conducted in two rural districts of Sindh province, Pakistan as part of a stunting prevention project between May and August 2014. A standard questionnaire on IYCF practices recommended by World Health Organization was used to collect information from 2013 mothers who had a child aged between 0 and 23 months.

**Results:**

Only 49% of mothers initiated breastfeeding within one hour of birth. Thirty-seven percent of mothers exclusively breastfed their infants for six months. Seventy-percent mothers introduced complementary feeding at 6–8 months of age. Eighty-two percent of mothers continued breastfeeding for at least one year and 75% for at least two years of age. IYCF practices were not significantly different for boys and girls in the study area. Being an employed mother (AOR 2.14; 95% CI 1.02, 4.51) was positively associated with the early initiation of breastfeeding. Children who were born at a health facility (AOR 0.65; 95% CI 0.50, 0.84) and were aged six to eleven months (AOR 0.70; 95% CI 0.54, 0.90) were less likely to be have an early initiation of breastfeeding. Mothers aged 25 to 29 years (AOR 1.83; 95% CI 1.05, 3.18), being literate (AOR 1.79; 95% CI 1.15, 2.78), and higher income (AOR 10.6; 95% CI 4.40, 25.30) were more likely to have an improved dietary diversity. Being an employed mother (AOR 2.18; 95% CI 1.77, 4.03) and higher income were more likely to have minimum acceptable diet (AOR 9.7; 95% CI 4.33, 21.71).

**Conclusion:**

IYCF practices were below the acceptable level and associated with maternal age, maternal illiteracy, unemployment, and poor household wealth status. Emphasis should be given to improve maternal literacy and reduction in poverty to improve IYCF practices.

## Background

In 1998, the executive board of the World Health Organization (WHO), called for the revitalization of the global commitment to appropriate infant and young child nutrition, and in particular breastfeeding and complementary feeding [1]. The first two years of the child’s life provide a critical window of opportunity to ensure survival, growth and development through optimum infant and young child feeding (IYCF) practices [2]. Optimal breastfeeding practices, including the initiation of breastfeeding within one hour of birth, exclusive breastfeeding (EBF) for six months [3], and continuation of breastfeeding for up to two years and beyond, with age appropriate complementary feeding, all have great potential for reducing under five malnutrition and thereby affecting child mortality rate [2, 4, 5].

The WHO therefore has recommended tracking IYCF indicators on the introduction of soft, solid or semi-solid foods, minimum dietary diversity, minimum meal frequency, and minimum acceptable diet during two years of age [6]. Early introduction of complementary feeding is a common cultural practice in South Asian countries including Pakistan [7–10], and the same counties contribute significantly to the high burden of under-nutrition in the region [9, 11]. The Pakistan National Nutrition Survey (NNS) 2011 reported in Sindh province that 51% of mothers initiated breastfeeding within one hour after birth, 67% predominantly maintained breastfeeding, and 9.6% of children were exclusively breastfed up to six months [12]. Similarly, 3.2% of children age 6–23 months of age were offered the minimum dietary diversity, 54.1% offered the minimum meal frequency, and 5.8% were offered an acceptable diet [13].

Sociodemographic factors associated with EBF included maternal age, education [13–15], income and health facility births [16, 17]. The mother’s formal education [18–20], and birth order of index child was significantly associated with early initiation of breastfeeding [20, 21].

Evidence suggests that maternal education is associated with timely introduction of complementary feeding, meal frequency, dietary diversity, and the practice of a minimum acceptable diet [22–25]. Moreover, household wealth status, geographical location, exposure to media, maternal age, and the utilization of antenatal and postnatal visits are associated with improved complementary feeding practices [22, 25–28]. Additional information is required to provide more evidence to monitor progress at the local level. This study was aimed to measure IYCF indicators and to identify factors associated with poor IYCF practices. The findings of this study will help policy makers to target behavior change strategies in mothers to enhance the IYCF practices in Pakistan.

## Methods

### Study design

A cross-sectional survey was conducted to collect data between May and August 2014. The survey was designed to provide IYCF and baseline indicators on a representative sample of households in the study area.

### Study setting

This study was conducted in two districts of Thatta and Sujawal in Sindh, Pakistan. These districts are administratively subdivided into nine Talukas and 55 union councils (UCs) with a population of 1.5 million. More than 88% of the population in Thatta and Sujawal has access to improved sources of drinking water. Sixty eight percent have access to electricity and 31% of households own some agriculture land. Finished floors are present in 35% of households, roofing in 36% and walls in 40% of households [29].

### Study participants

The study participants for this survey were the mothers of children aged less than two years who lived in the study area for more than six months. In the case of having two or more mothers with children under-two years of age in the household, we selected the one with younger child.

### Sample size

Sample size for this study was calculated using comparison of two sequential surveys approach. This design was used to assess the impact of intervention over time as discussed in Micronutrient Initiative (MI) and Center for Disease Control (CDC) manual [30]. Sample size calculation considered the prevalence of stunting in Sindh at 55% (NNS 2011) and design effect of 1.5. Total sample size was 2166 per survey to detect 10% difference in stunting. With a power of 80% and a significance level of 5%, the sample size was inflated by 10% for non-response.

### Sampling methodology

A two-staged cluster sampling technique was used to select households for interviews. In the first stage, the sample size was divided in 29 UCs using a Proportion to Population Size (PPS) design. In the second stage, the villages and households with children under-two years of age were randomly selected for interview on baseline and IYCF indicators.

### Data collection

Sixteen data collectors, four team leaders, two desk editors and two field supervisors were trained and involved in baseline data collection. Senior faculty and staff belonging to the Department of Paediatrics and Child Health, Aga Khan University arranged the five day training. Three days were dedicated to didactic training of staff, one day was spent on field pilot testing and one day for a short refresher that included problems and irregularities found during the pilot testing.

A standard questionnaire on IYCF practices recommended by WHO was used to collect information from mothers with children aged between 0 and 23 months. The information collected included IYCF practices and socioeconomic characteristics. The questionnaire was initially designed in English, with subsequent translation to Sindhi by a senior project team staff that was well versed in both languages. It was then translated back in English. An independent review of the questionnaire was done eventually to check for any inconsistencies.

### Definition of variables used in the study

#### Early initiation of breastfeeding

Children born in the last 24 months who received breast milk within one hour of birth.

#### Exclusive breastfeeding

Infants 0–5 months of age who received only breast milk and nothing else: no other milk, food, drink, not even water during the previous day.

#### Continued breastfeeding at one year

Children 12–15 months of age who received breast milk during the previous day.

#### Introduction of solid, semi-solid or soft foods

Infants 6–8 months of age who received solid, semi-solid or soft foods during the previous day.

#### Minimum dietary diversity

Children 6–23 months of age who received foods from four or more food groups during the previous day.

#### Minimum meal frequency

Breastfed and non-breastfed children 6–23 months of age who receive solid, semi-solid, or soft foods (but also including milk feeds for non-breastfed children) the minimum number of times or more during the previous day.

#### Minimum acceptable diet

Children 6–23 months of age who receive a minimum acceptable diet (apart from breast milk) during the previous day.

#### Consumption of iron-rich or iron-fortified foods

Children 6–23 months of age who received an iron-rich food or a food that was specially designed for infants and young children and was fortified with iron, or a food that was fortified in the home with a product that included iron during the previous day.

#### Continued breastfeeding at two years

Children 20–23 months of age who received breast milk during the previous day.

#### Age-appropriate breastfeeding

Infants 0–5 months of age who received only breast milk during the previous day, and children 6–23 months of age who received breast milk, as well as solid, semi-solid or soft foods, during the previous day.

#### Predominant breastfeeding under six months

Infants 0–5 months of age who received breast milk as the predominant source of nourishment during the previous day.

#### Taluka

Taluka or Tehsil is second administrative level after district in all provinces of Pakistan.

#### Union council

Union council is lowest administrative unit in Pakistan with a population between 25,000 to 70,000.

### Statistical analysis

Paper based data was entered on predefined data entry screens using Microsoft visual fox pro version 9.0. STATA version 12 was used for data analysis. Frequencies and percentages were calculated for categorical variables. Multivariable analysis was used for the impact of each variable by odds ratio with 95% CI and *p* - value <0.05 being considered statistically significant. Initiation of breastfeeding, EFB, minimum dietary diversity, minimum acceptable diet and age appropriate breastfeeding were defined as binary variables. We tested bivariate analysis for factors associated with all outcome variables. The *p* - value <0.25 in the bivariate was used for inclusion in the multivariable logistic regression model. Factors that were insignificant at the multivariable model were excluded after careful assessment of confounders. The final model was selected on the basis of theoretical and statistical significance of predictors. Clusters were adjusted to take sampling design into account.

Wealth indexes were based on the principal component analysis using household assets, household ownership status, materials used for the roof, floor and wall of the house, number of rooms, fuel for cooking, main source of drinking water and toilet facility. Wealth scores were divided into five parts to make wealth quintile: poorest, poor, middle, rich and richest [31].

## Results

### Characteristics of study sample

All mothers in the selected households consented to participate in the survey. A total of 2013 mother-child pairs were included for analysis. The mean age of mothers was 30 years (SD ± 0.25 years) and mean age of children was 11 months (SD ± 0.12 months). The majority of mothers (87%) were illiterate. A very small proportion (4%) of mothers was employed, while 96% were a homemaker. Sixty-two percent received 1–3 outpatient antenatal care visits with a primary care provider, while 20% didn’t receive any antenatal care during their last pregnancy. Approximately 58% were health facility births. The gender proportion was almost equal (50.7% and 49.3%). Improved drinking water was available in 38% of the households. Forty-one percent of households were using improved toilet facilities. Four percent of households were severely food insecure, approximately half of households were moderately food insecure and 46% of households experienced none or light hunger (Table 1).

**Table 1:** Characteristics of study sample

Sociodemographic variables	*n* = 2013	
Age of mothers	*n*	%
< 25 Years	312	15.5
25–29 Years	669	33.2
30–35 Years	633	31.4
36 + Years	399	19.8
Mean ± SD	30.3 ± 0.25	
Educational status of mothers		
Illiterate	1747	86.8
Literate	266	13.2
Occupation of mothers		
Employed	81	4.0
Housewife	1932	96.0
ANC visits during last pregnancy		
None	411	20.4
1–3 visits	1250	62.1
4+ visits	352	17.5
Place of birth		
At health facility	1167	58.0
At home	846	42.0
Age of children		
0–5 months	587	29.2
6–11 months	626	31.1
12–23 months	800	39.7
Mean ± SD	10.6 ± 0.12	
Sex of child		
Male	1021	50.7
Female	992	49.3
Source of drinking water		
Improved	767	38.1
Unimproved	1246	61.9
Toilet facility		
Improved	834	41.4
Unimproved	1179	58.6
Household food insecurity		
None or light hunger (0–1 score)	585	46.5
Moderate hunger (2–3 scores)	623	49.5
Severe hunger (4–6 scores)	50	4.0
Wealth Index		
Poorest	410	20.4
Poor	391	19.4
Middle	402	20.0
Rich	403	20.0
Richest	407	20.2

### Infant and young child feeding practices

Table 2 presents infant and young child feeding practices in the study area. About half of mothers (49%) initiated breastfeeding within one hour of birth. More than quarter (37%) was exclusively breastfeeding up to six months, while 68% were predominantly breastfeeding under six months in the last 24 h preceding the survey. Seventy-percent of mothers introduced solid, semi-solid or soft foods during six to eight months of age. Only 38% of children (6–23 months) received the minimum meal frequency, 10% received the recommended minimum dietary diversity, and 8% received the minimum acceptable diet in the last 24 h preceding the survey. Almost all children (99.9%) were ever breastfed equally, both boys and girls. Eighty-two percent of mothers continued breastfeeding up to one year of age, while 75% continued up to two years of age. Age appropriate breastfeeding (0–23 months) was 78%. Overall 12% of mothers used bottle feeding (14% for boys & 10.4% for girls). Other IYCF practices for both genders were not remarkably different.

**Table 2:** IYCF practices for the children in the study sample

IYCF Practices	Overall*n* (%)	Boys*n* (%)	Girls*n* (%)	*p* - value
Early initiation of breastfeeding within 1 h	963 (48.8)	480 (47.9)	483 (49.7)	0.351
Exclusive breastfeeding under 6 months	217 (37.0)	105 (37.1)	112 (36.8)	0.942
Continued breastfeeding at 1 year	236 (82.5)	117 (79.1)	119 (86.2)	0.056
Introduction to solid, semi-solid or soft foods (6–8 months)	229 (70.0)	118 (69.0)	111 (71.2)	0.709
Minimum dietary diversity (6–23 months)	142 (9.9)	70 (9.5)	72 (10.5)	0.653
Minimum meal frequency (6–23 months)	545 (38.2)	275 (37.3)	270 (39.2)	0.389
Minimum acceptable diet (6–23 months)	112 (7.9)	53 (7.2)	59 (8.6)	0.414
Consumption of iron-rich or iron-fortified foods	360 (25.3)	178 (24.1)	182 (26.5)	0.297
Children ever breastfed	1985 (99.9)	1006 (99.9)	979 (99.9)	0.589
Continued breastfeeding at 2 years	133 (74.7)	72 (74.2)	61 (75.3)	0.875
Age-appropriate breastfeeding	1543 (77.7)	768 (76.3)	775 (79.2)	0.205
Predominant breastfeeding under 6 months	398 (67.8)	191 (67.5)	207 (68.1)	0.861
Bottle feeding	243 (12.3)	142 (14.1)	101 (10.4)	0.015

### Determinants of infant and young child feeding practices

Table 3 presents determinants of infant and young child feeding practices in the study area. Being an employed mother (AOR 2.14; 95% CI 1.02, 4.51) was positively associated with the early initiation of breastfeeding. Birthing at a health facility (AOR 0.65; 95% CI 0.50, 0.84) and children aged 6–11 months (AOR 0.70; 95% CI 0.54, 0.90) were negatively associated with early initiation of breastfeeding. Mothers who received between one to three antenatal care visits (AOR 0.55; 95% CI 0.32, 0.95) and delivered at health facility (AOR 0.64; 95% CI 0.43, 0.96) were less likely to practice EBF under six months. Mothers aged 25–29 years were more likely to offer the minimum dietary diversity as compared to mothers of other age groups (AOR 1.83; 95% CI 1.03, 3.26). Being an literate mother (AOR1.79; 95% CI 1.15, 2.78), mothers aged between 25 and 29 years (AOR 1.83; 95% CI 1.05, 3.18), and a higher income (AOR 10.6; 95% CI 4.40, 25.30) was positively associated with the minimum dietary diversity. Children aged six to eleven months (AOR 0.46; 95% CI 0.30, 0.70) were less likely to receive the minimum dietary diversity as compared to other children. It was found that mothers who were employed (AOR 2.18; 95% CI 1.77, 4.03), and belonged to richest wealth quintiles (AOR 9.7; 95% CI 4.33, 21.71) were more likely to provide the minimum acceptable diet to their children in the last 24 h than their counterparts. Children aged six and eleven months (AOR 0.50; 95% CI 0.31, 0.79) were less likely to receive the minimum acceptable diet as compared to other children. Mothers aged between 30 and 35 years (AOR 1.59; 95% CI 1.11, 2.26) were more likely to practice age appropriate breastfeeding as compared to other mothers. Children under six months (AOR 3.28; 95% CI 2.11, 5.09) were more likely to receive age appropriate breastfeeding as compared to older children. The middle (AOR 0.51; 95% CI 0.36, 0.72) and richest wealth quintiles (AOR 0.50; 95% CI 0.32, 0.77) were less likely to receive age appropriate breastfeeding compared to other wealth quintiles.

**Table 3:** Factors associated with infant and young child feeding practices

Variables	Early initiation of breastfeeding within 1 h		Exclusive breastfeeding under 6 months		Minimum dietary diversity		Minimum acceptable diet		Age-appropriate breastfeeding	
	Adjusted OR 95% CI	*p*-value	Adjusted OR 95% CI	*p*-value	Adjusted OR 95% CI	*p*-value	Adjusted OR 95% CI	*p*-value	Adjusted OR 95% CI	*p*-value
Maternal age										
< 25 Years					1				1	
25–29 Years					1.83 (1.05, 3.18)	0.032			1.09 (0.76, 1.50)	0.634
30–35 Years					1.56 (0.93, 2.59)	0.088			1.59 (1.11, 2.26)	0.010
36 + Years					0.91 (0.42, 1.97)	0.818			1.48 (0.97, 2.24)	0.066
Maternal education										
Illiterate					1					
Literate					1.79 (1.15, 2.78)	0.009				
Maternal occupation										
Employed	2.14 (1.02, 4.51)	0.043					2.18 (1.77, 4.03)	0.013		
Housewife	1						1			
ANC visits during last pregnancy										
None			1							
1–3 visits			0.55 (0.32, 0.95)	0.032						
4 + visits			0.7 (0.33, 1.46)	0.348						
Place of birth										
Health facility	0.65 (0.50, 0.84)	0.001	0.64 (0.43, 0.96)	0.031						
Home	1		1							
Child age										
0–5 months	0.92 (0.74, 1.15)	0.501							3.28 (2.11, 5.09)	<0.001
6–11 months	0.70 (0.54, 0.90)	0.007			0.46 (0.30, 0.70)	<0.001	0.50 (0.31, 0.79)	0.003	0.71 (0.54, 0.93)	0.015
12–23 months	1				1		1		1	
Wealth quintiles										
Poorest					1		1		1	
Poor					3.18 (1.08, 9.32)	0.036	2.57 (0.84, 7.79)	0.096	1.06 (0.70, 1.57)	0.792
Middle					3.38 (1.10, 10.39)	0.033	2.88 (0.88, 9.32)	0.078	0.51 (0.36, 0.72)	<0.001
Rich					4.92 (1.93, 12.47)	0.001	4.27 (1.76, 10.30)	0.001	0.78 (0.52, 1.16)	0.225
Richest					10.60 (4.40, 25.30)	<0.001	9.7 (4.33, 21.71)	<0.001	0.50 (0.32, 0.77)	0.002

## Discussion

The primary objective of this study was to assess IYCF practices and to identify potential determinants of the persistent low rates of IYCF practices in two rural districts of Sindh, Pakistan. Our results revealed that the IYCF practices were suboptimal. Only about half of mothers in our study reported that they initiated breastfeeding within one hour of birth. The prevalence of EBF and predominant breastfeeding was (37% and 68%), higher than that reported (29% and 56%) in Multiple Indicator Cluster Survey (MICS) Sindh 2014 [29], and a study conducted in Gilgit Pakistan [32]. This may be attributed to the difference in sampling strategies. The prevalence of EBF in our study was similar with the national rates reported in PDHS 2006–07 [33], 2012–13 [31] and in studies conducted in developing countries [34, 35].

Almost all (99.9%) of mothers in our study had ever practiced breastfeeding which is almost similar to the recent Sindh provincial rates [31] and Dera Ghazi Khan in Pakistan [36]. Similar studies conducted in Ethiopia and Nepal [37–39] show comparative results. Seventy-percent mothers introduced solid, semi-solid or soft foods during six to eight months of age and this high frequency is similar with Sindh provincial rates reported in MICS 2014 report [29], and studies in Nigeria [40, 41].

The proportion of infants receiving recommended minimum dietary diversity (10%), minimum meal frequency (38%) and minimum acceptable diet (8%) was much lower in our study setting. A multi-country study conducted in five South Asian countries, including Pakistan, reported children aged 6–23 months received the minimum dietary diversity that ranged from a minimum of 15% in India to a maximum 71% in Sri Lanka [27]. However, the proportion of infants receiving recommended minimum dietary diversity and minimum acceptable diet in our study setting was comparable with provincial rates [29] but lower than other studied conducted in developing countries [27, 40, 42]. This disparity may be related to the accessibility and availability to specific foods such as rice, wheat, potato and fish or the cultural dependency on food items that are deficient on important nutrients.

Evidence from Asian countries reveals that education of the mother is significantly associated with timely initiation of complementary foods and the minimum acceptable diet. Maternal education was also positively associated with infant feeding in other studies [22, 24, 43] but our study only showed association with minimum dietary diversity (AOR 1.79; 95% CI 1.15, 2.78). This may be a reflection of our poor education system that does not provide an adequate and quality education about nutrition.

We found a significant association of a minimum dietary diversity (AOR 10.6; 95% CI 4.40, 25.30), the minimum acceptable diet (AOR 9.70; 95% CI 4.33, 21.71) and age appropriate breastfeeding (AOR 0.50; 95% CI 0.32, 0.77) with the richest wealth quintiles. Our study is one of the few studies conducted in rural districts of Sindh that allowed the WHO guidelines on data collection for assessing IYCF indicators.

The study had some important limitations. To begin with, we cannot generalize it to every rural setting due to the small sample size. Moreover, due to the cross-sectional study design, conclusions on the cause-effect relationship cannot be drawn. We were also not able to include the effect of seasonal variation and cultural practices on food availability and food consumption patterns. However it does provide a critical insight to the IYCF practices and some of the indicators that policy makers can focus upon.

## Conclusions

The findings of our study reveal that the current IYCF practices are poor and significantly associated with maternal age, maternal illiteracy, unemployment, and poor household wealth status. Thus, emphasis should be given to improve maternal education status and employment for mothers. Moreover, special attention should be given to mothers with poor socioeconomic status to improve IYCF practices. Further research is recommended to investigate the potential factors associated with child feeding practices and interventions, to improve IYCF indicators in children under-two years of age.

## Abbreviations

CDC: Center for Disease Control
ERC: Ethical Review Committee
IYCF: Infant and Young Child Feeding
MICS: Multiple Indicator Cluster Survey
NBC: National Bio-Ethics Committee
NNS: National Nutrition Survey
PPS: Proportion to Population Size
UC: Union Council
WHO: World Health Organization
